# Experimental addition of marine-derived nutrients affects wildflower traits in a coastal meta-ecosystem

**DOI:** 10.1098/rsos.221008

**Published:** 2023-01-25

**Authors:** Allison M. Dennert, E. Elle, John D. Reynolds

**Affiliations:** Department of Biological Sciences, Simon Fraser University, Burnaby, BC, Canada V5A 1S6

**Keywords:** marine-derived nutrients, meta-ecosystem, floral traits, nitrogen, subsidy

## Abstract

Organismal movement can bring individuals, resources and novel interactions across ecosystem boundaries and into recipient habitats, thereby forming meta-ecosystems. For example, Pacific salmon ecosystems receive large marine-derived nitrogen subsidies during annual spawning events, which can have a wide range of effects on aquatic and terrestrial plant species and communities. In this study, we evaluate the effects of cross-ecosystem nutrient subsidies on terrestrial plant growth and reproduction. We conducted a large-scale field experiment with four treatments: (i) addition of a pink salmon (*Oncorhynchus gorbuscha*) carcass, (ii) addition of the drift seaweed rockweed (*Fucus distichus*), (iii) addition of both salmon + rockweed, and (iv) a control. We examined treatment effects on leaf nitrogen and fitness-associated floral traits in four common estuarine wildflower species. We found elevated leaf ∂^15^N in all plant species and all sampling years in treatments with salmon carcass additions but did not observe any differences in leaf per cent nitrogen. We also observed larger leaf area in two species, a context-dependent increase in floral display area in two species, and a limited increase in plant seed set in response to both salmon carcass treatments. In sum, our study suggests that marine nutrients can affect terrestrial plant growth and reproduction.

## 1. Introduction

Meta-ecosystems are defined as sets of ecosystems connected by organisms, energy and materials [1,2]. Movement of plants and animals via dispersal, foraging or migration can bring individuals, resources and novel species interactions to recipient habitats across ecosystem boundaries [3,4]. Life cycle migration—often characterized by organismal death after mating—may bring large cross-ecosystem resource subsidies to recipient ecosystems and is a key component of meta-ecosystem theory [2].

The meta-ecosystem is a useful framework in coastal rivers, particularly at land–sea interfaces where anadromous and semelparous fishes spawn and die during their life cycle migrations [2]. Anadromous Pacific salmon provide organisms, energy and materials as they return to their natal streams to spawn [5–7]. They provide novel organismal interactions through bioturbation and egg competition prior to their deaths [8–10], energy via altered ecosystem metabolic properties [11] and nutrient subsidies through their carcasses to both aquatic and terrestrial habitats [5,12,13]. In this way, salmon ecosystems reflect the meta-ecosystem concept.

Marine-derived resource subsidies in salmon ecosystems have terrestrial effects that scale from communities to individuals [14]. Effects on terrestrial communities include the alteration of plant, insect, and bird community structure and diversity [15–17]. Effects on individuals can be seen in birds, where salmon subsidies can influence male habitat selection, behaviour and territory size [18], and riparian plants, where salmon subsidies can increase stomatal density and fruit production [19,20]. Much of the recent work on salmon subsidies in terrestrial habitats has been necessarily observational, with a few notable exceptions [21–23]. A potentially powerful and complementary approach to observational studies is to test experimentally for effects of carcass deposition on individual plants living in salmon meta-ecosystems. Particularly in an experimental and causal context, the extent to which nutrient subsidies from salmon influence terrestrial plant traits involved in growth and reproduction is not well understood.

In this study, we test experimentally for effects of cross-ecosystem nutrient subsidies on growth and reproduction of terrestrial plants. We conducted a multi-year, large-scale field experiment in a saltmarsh wildflower meadow on the banks of an estuary on the central coast of British Columbia, Canada. We sought to test whether and how marine-derived nutrients from Pacific salmon carcasses and marine drift seaweed affect the following fitness-associated traits in four common wildflowers: leaf area, inflorescence size and seed production. We also tested whether the addition of marine-derived nutrients affected the nitrogen isotope ratios and per cent nitrogen in the leaf tissues of these plants. We were interested in examining drift seaweed, as sea wrack is a common source of macro- and micro-nutrient deposition at land–sea interfaces and is ubiquitous in our study area [24,25]. Additionally, drift seaweed may provide different amounts of elemental nutrients than salmon carcasses—such as calcium, magnesium, potassium and phosphorus—whereas salmon carcasses primarily provide marine-derived nitrogen. We predicted that the addition of marine organisms to this wildflower meadow would supplement limiting nutrients to these plant species, and that we would see changes in plant traits in our experimental treatments, such as an increase in leaf area or floral display size. We hypothesize that the limiting nutrients provided by salmon and drift seaweed may differ, and result in differential responses in plant traits between subsidy types. The traits and architecture of primary producers can regulate ecosystem processes, create habitat and control resources for other taxa [26]. Thus, plants are the interface between the biotic and abiotic worlds and provide a wide variety of traits to test for evidence of cross-ecosystem resource subsidies in the context of meta-ecosystem ecology. We aim to test the importance of marine-derived nutrients to terrestrial plant traits involved in growth and reproduction, thus providing an empirical example of meta-ecosystem level processes that may affect individual organisms.

## 2. Methods

### 2.1. Study site

**Figure 1.**
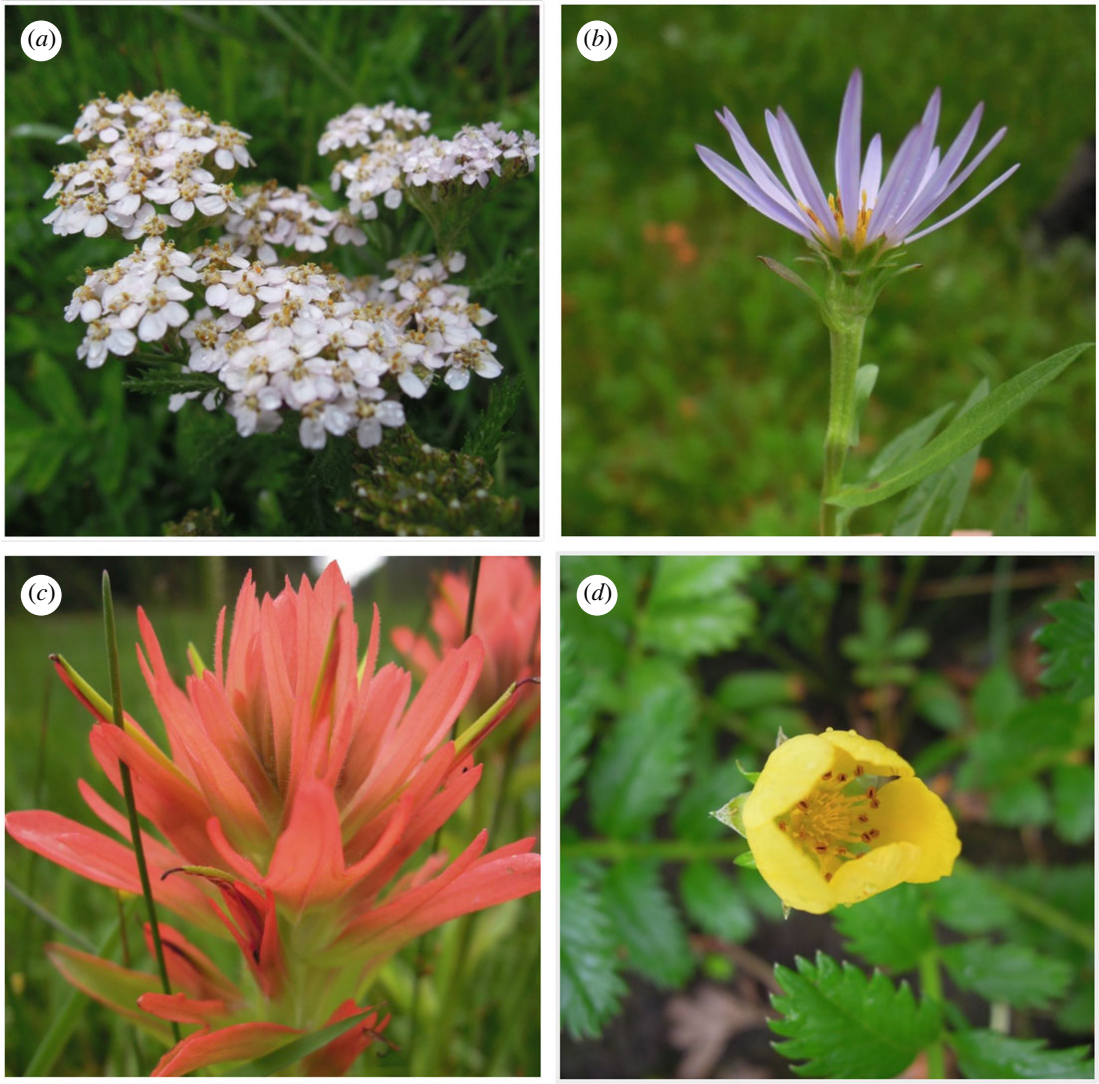
The four focal plant species: (*a*) yarrow (*Achillea millefolium*, Asteraceae), (*b*) Douglas' aster (*Symphyotrichum subspicatum*, Asteraceae), (*c*) common red paintbrush (*Castilleja miniata*, Orobanchaceae) and (*d*) silverweed (*Potentilla anserina*, Rosaceae).

We conducted this experiment in the estuary of a small river on the central coast of British Columbia, Canada, in the territory of the Haíɫzaqv (Heiltsuk) First Nation. The estuary is located approximately 18 km west of Wágl̇ísl̇a (Bella Bella), BC (52°12′41.8″ N, 127°52′49.8″ W, electronic supplementary material, figure S1). The river that flows into the estuary receives an average of less than 200 spawning salmon annually (Reynolds Lab, unpublished data), and the stream geomorphology prevents the deposition of large amounts of drift seaweed onto the banks of the study area. The study watershed is small, with a catchment area of 4.5 km^2^, but features a relatively large estuary meadow populated with grasses, sedges and salt-tolerant wildflowers. These wildflower assemblages are dominated by silverweed (*Potentilla anserina*, Rosaceae), yarrow (*Achillea millefolium*, Asteraceae), Douglas' aster (*Symphyotrichum subspicatum*, Asteraceae), common red paintbrush (*Castilleja miniata*, Orobanchaceae), springbank clover (*Trifolium wormskjoldii*, Fabaceae) and sea watch (*Angelica lucida*, Apiaceae). We selected the four most common wildflower species in each plot (figure 1; silverweed, common red paintbrush, Douglas’ aster, yarrow) as focal plant species to address our research questions. The study area is accessible only by boat, and human disturbance is minimal. This region is in the Coastal Western Hemlock Biogeoclimatic zone, which is characterized by a maritime climate with moderate temperatures and heavy rainfall (greater than 3000 mm annually). Summer season temperatures range from an average low of 9°C to a, average high of 21°C, with an average of 12–14 days of rain per summer month.

### 2.2. Experimental design

We introduced salmon carcasses and marine algae into the estuary in October 2016, 2017 and 2018 using a blocked factorial design (electronic supplementary material, figure S2), which features a control and three treatments with 25 replicates per treatment (*N* = 100): (i) an addition of a pink salmon (*O. gorbuscha*) carcass, (ii) an addition of the drift seaweed rockweed (*Fucus distichus*), (iii) an addition of both salmon + rockweed, and (iv) a control with no additions. Salmon carcass mass was limited to 1–1.5 kg, while rockweed additions were approximately 250 g. Each 1 m^2^ plot was placed 1 m away from adjacent plots in the same block; experimental blocks were at least 3 m apart from water sources and from each other (electronic supplementary material, figure S3). Prior experimental [21] and observational [27] work has shown that the effect of salmon carcasses on soil nutrients is a transient and heterogenous effect. By using a 1 m^2^ plot, we may detect the direct consequences of an individual carcass, which allows for calculations in terms of transferable measurement units (e.g. organisms, m^2^) that can scale from individuals to ecosystems—a need identified by prior reviews in the meta-ecosystem literature [2], as well as in a recent comprehensive review of impacts of salmon around the North Pacific [14]. Roots of clonal species that may run between plots were cut at least twice annually to a depth of 30 cm using a machete (electronic supplementary material, figure S2, dotted line), after we ensured during exploratory surveys that the maximum root and rhizome depth for all species was 23 cm. Cuts were made in between the plots in each block, 50 cm away from the centre plot boundaries (electronic supplementary material, figure S2). Rooting depth and spread is strongly correlated to plant height, and on average the rooting spread of perennial grasses and forbs is less than 1 m [26,28]. We placed salmon and/or rockweed into a mesh bag and staked them into the plots to prevent displacement via wind or occasional flooding. We added empty, staked mesh bags to the control plots. We could not identify a viable method of control for inevitable animal scavenging, though we did monitor the plots for multiple weeks post-treatment to ensure that the carcasses remained in place and in some cases replaced carcasses that had been disturbed by wildlife.

### 2.3. Stable isotope analysis

To examine whether the salmon-derived nutrients from the carcass additions remained within each plot and were taken up by each plant species, each year at the end of July we haphazardly collected one leaf from each of the four focal plant species present in each plot and placed them into paper coin envelopes for storage. Samples were then dried at 60°C for a minimum of 48 h before they were ground into a fine powder using a Wig-L-Bug grinder. We then packaged up to approximately 4.6 mg of each sample into aluminium capsules for analysis. An elemental analyser with isotope ratio mass-spectrometer (EA-IRMS) was used to calculate isotopic ratios of nitrogen, which is expressed in isotopic ratios of nitrogen and abbreviated with delta notation, *δ*. Isotopic signature calculations used the following formula: *δ*^15^N(‰) = (*R*_sample_/*R*_standard_ − 1) × 1000, where *R* indicates the ratio of ^15^N to ^14^N.

### 2.4. Focal trait estimates

To quantify the effect of treatment on vegetative and reproductive plant traits, we produced estimates of three characteristics: leaf area, floral display area and seed production. We selected these three focal traits as they provide a simple means to measure responses in plant vegetative growth, reproductive allocation and reproductive success, respectively. Additionally, given the differing morphology of our study species, these traits allowed us to conduct the most consistent and transferable methods across species. First, we tagged up to three individuals of the four focal plant species in each treatment plot. We obtained measures for both leaf area and floral display area during peak bloom of each species, or when approximately 40–60% of inflorescences were in bloom. To estimate leaf area, we selected a random leaf on the tagged plant, measured the leaf length and width in mm, and used the product of these measurements and the total number of leaves on the tagged plant. Next, to estimate floral display area, we measured the number and size of all inflorescences produced by each tagged plant. If the flower was large, round and easily counted individually (e.g. silverweed), we counted and measured the corolla diameter of all flowers from the focal plant. If the plant had an inflorescence composed of few flowers without a round corolla (e.g. common red paintbrush), we measured the diameter of the entire inflorescence. If the plant was in the family Asteraceae (e.g. Douglas' aster, yarrow), we counted and measured all inflorescences separately. Using the diameter of the inflorescences, we calculated the approximate circular display area of each inflorescence and added the area from all inflorescences to estimate floral display area.

Finally, to quantify seed production, we enclosed the inflorescences of each tagged focal plant with mesh bags at the end of the bloom period and left them for a minimum of 7 days for seeds to mature. We then collected the bagged inflorescences and used dissecting and compound microscopes to count the number of fertile seeds produced by each flower. Silverweed and Douglas’ aster seeds were counted in full. Yarrow and common red paintbrush seeds were subsampled and seed number was estimated due to the large volume of seeds produced by these species. To subsample yarrow, we dissected six florets for seed counts. We then multiplied the average number of seeds per dissected floret by the total number of florets on the inflorescence. To subsample common red paintbrush, we dissected and counted seeds from three capsules and multiplied the average number of seeds per capsule by the total number of flowers on the inflorescence.

### 2.5. Statistical analyses

We conducted all statistical analyses in R. We created four statistical models to assess the effect of treatment (salmon, rockweed, both salmon + rockweed or control) on our four response variables (isotope ratios, leaf size, flower size and seed set), while accounting for plant species and year. To explore isotope ratios, we created a linear mixed effects model in the lme4 package [29], as the data were continuous and normally distributed. To examine our three plant characteristics, we used generalized linear mixed effects models with nested random effects in the glmmTMB package [30] to account for multiple plants measured in each treatment. Each model describes the response variable (*δ*^15^N, leaf area, floral display area and seed set) as a function of treatment group, plant species and year, while allowing only for two-way interactions between the three predictor variables. The random effect structure for the plant characteristic models nests individual plot within block, whereas the random effect structure for nitrogen-15 models contains only block, due to differences in the number of plants collected or measured within each treatment plot. For each model, we used the DHARMa package [31] to verify residual normality and variance homogeneity, check for outliers, reduce overdispersion and verify model fit.

Due to the categorical nature of our model parameters and their interactions, it was difficult to use model coefficient estimates alone to communicate the magnitude of effect of treatment, plant species and year on our response variables. Thus, we calculated model predictions for each level of our categorical variables with least-squares means and standard errors using the emmeans package [32]. Comparisons with the control treatment for each species within each year were done using *post hoc* Dunnett-adjusted *p*-value comparisons (see electronic supplementary material, tables S1–S4).

## 3. Results

### 3.1. Stable isotopes

**Figure 2.**
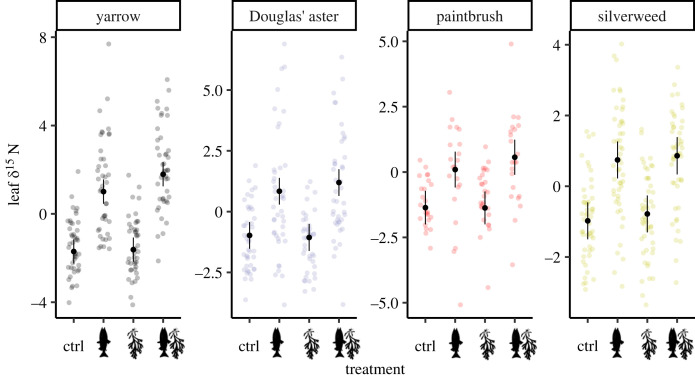
Leaf *δ*^15^N in relation to the four treatments in the four plant species. Points indicate the raw data overlaid by the modelled least-squares means with 95% confidence intervals in black, averaged across years (*N* = 629).

We observed elevated ∂^15^N in the leaves of all focal plant species in all sampling years in the two treatments with salmon carcasses (figure 2; electronic supplementary material, table S1; modelled least-squares means for each treatment pooled across species and year: control: −1.255‰ ± 0.210; salmon: 0.671‰ ± 0.213; rockweed: −1.209‰ ± 0.209; salmon + rockweed: 1.104‰ ± 0.212; *N* = 629). While this pattern was consistent within all three sampling years and within all four species, the difference between the control and carcass treatments was largest in yarrow. Leaf per cent nitrogen varied by species, but there were no differences between any of the four treatments in any of the three sampling years (electronic supplementary material, figure S4; modelled least-squares means for each species pooled across treatment and year: yarrow, 2.01% ± 0.03; Douglas' aster, 2.22% ± 0.03; common red paintbrush, 1.67% ± 0.04; silverweed, 2.04% ± 0.03; *N* = 629). Common red paintbrush had the lowest leaf ∂^15^N and per cent nitrogen of any species, irrespective of treatment and sampling year. Full Dunnett-adjusted comparisons with the control within species and sampling years are in the electronic supplementary material, table S1.

### 3.2. Focal traits—leaf area

**Figure 3.**
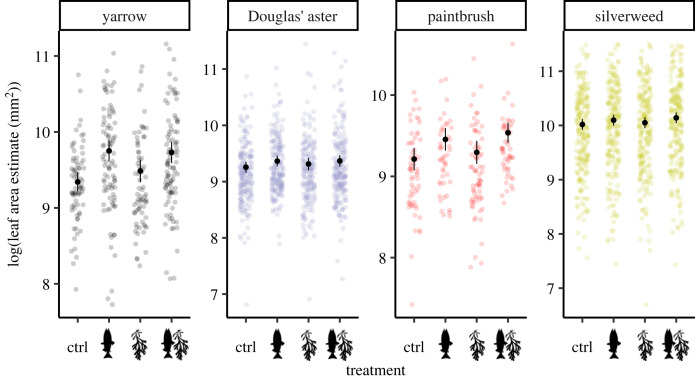
The logarithm of the estimated leaf area (mm^2^) in relation to the four treatments in the four plant species. Points indicate the raw data in link space overlaid by the modelled least-squares means with 95% confidence intervals in black, averaged across years (*N* = 2180).

Effects of the treatments on leaf area depended upon species and sampling year. In yarrow, the two treatments with salmon carcasses had the largest leaf area in all three sampling years (figure 3; electronic supplementary material, table S2; *N* = 2180). In Douglas’ aster, there were no discernable differences in leaf area estimates between any of the four treatments within any of the three sampling years (electronic supplementary material, table S2). In common red paintbrush, leaf area was larger in the two treatments with salmon carcasses than in the control treatment in all of the sampling years, with the exception of the salmon-only carcass treatment in 2018 which did not differ from the control in this species and year (Dunnett-adjusted *p* = 0.215; d.f. = 2100). Lastly, in silverweed, there were no differences in leaf area estimates from the control in any of the sampling years, with the exception of the salmon + rockweed treatment in the third sampling year, which had a larger leaf area than the control in this species and year (Dunnett-adjusted *p* = 0.050; d.f. = 2100). Full comparisons within species and sampling years are in the electronic supplementary material, table S2.

### 3.3. Focal traits—floral display area

**Figure 4.**
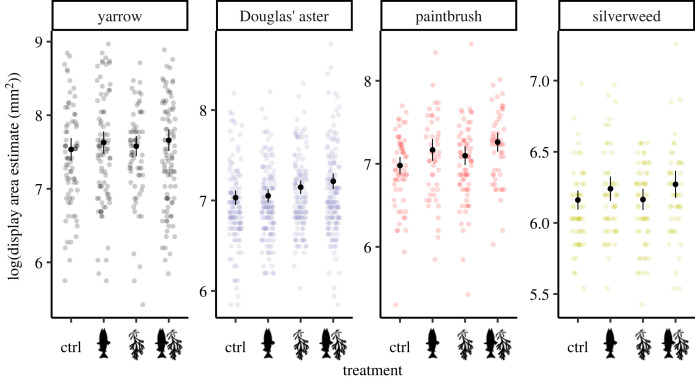
The logarithm of the estimated floral display area (mm^2^) in relation to the four treatments in the four plant species. Points indicate the raw data in link space overlaid by the modelled least-squares means with 95% confidence intervals in black, averaged across years (*N* = 1337).

In yarrow and silverweed, there were no differences in floral display size between any of the treatments within any of the sampling years (figure 4; electronic supplementary material, table S3; *N* = 1337). In Douglas' aster, there were variable patterns in display size in response to the treatments across years. In 2017, the rockweed treatment (Dunnett-adjusted *p* = 0.011; d.f. = 1257) and the salmon + rockweed treatment (Dunnett-adjusted *p* = 0.032; d.f. = 1257) had larger display sizes than the control. In 2018, only the salmon + rockweed treatment was significantly larger than the control (Dunnett-adjusted *p* = 0.039; d.f. = 1257). In 2019, both the rockweed treatment and the salmon + rockweed treatment had larger display sizes than the control (Dunnett-adjusted *p* = 0.048 and 0.014, respectively; d.f. = 1257). In common red paintbrush, only the salmon + rockweed treatment had a larger display size than the control in 2017 (Dunnett-adjusted *p* = 0.006; d.f. = 1257) and 2018 (Dunnett-adjusted *p* = 0.009; d.f. = 1257). In 2019, both the salmon treatment and the salmon + rockweed treatment had larger display sizes than the control (Dunnett-adjusted *p* = 0.018 and 0.003, respectively; d.f. = 1257). Full Dunnett-adjusted comparisons within species and sampling years are in the electronic supplementary material, table S3.

### 3.4. Focal traits—seed set

**Figure 5.**
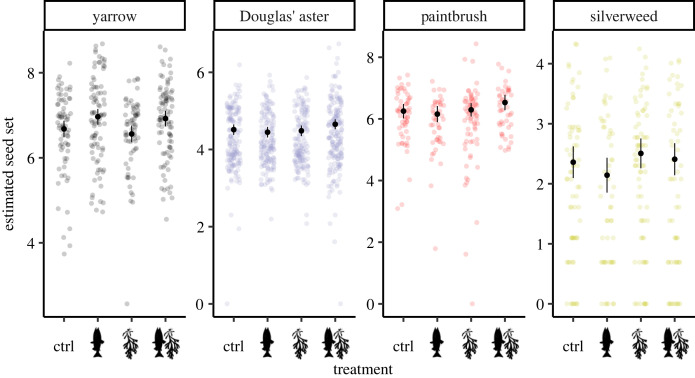
Estimated seed set in relation to the four treatments in the four plant species. Points indicate the raw data in link space overlaid by the modelled least-squares means with 95% confidence intervals in black, averaged across years (*N* = 1458).

There was no difference in seed set in response to any of the treatments in three of the plant species: Douglas’ aster, silverweed and common red paintbrush (figure 5; electronic supplementary material, table S4; modelled least-squares means for each treatment pooled across species and year: control, 146.94 seeds ± 1.07; salmon: 151.41 seeds ± 1.07; rockweed: 142.59 seeds ± 1.07; salmon + rockweed: 174.16 seeds ± 1.07; *N* = 1458). However, in 2019, yarrow produced more seeds in the salmon treatment (Dunnett-adjusted *p* = 0.025, d.f. = 1411) and the salmon + rockweed treatment (Dunnett-adjusted *p* = 0.024, d.f. = 1411) than in the control, but not in 2017 or in 2018. Full Dunnett-adjusted comparisons within species and sampling years are in the electronic supplementary material, table S4.

## 4. Discussion

This field experiment indicates that marine-derived nutrients have context-dependent effects on plant traits, which vary by plant species and through time. Although we found a strong effect of salmon carcass treatments on leaf nitrogen-15, there was no effect of treatment on leaf per cent nitrogen for any species. Furthermore, some species had larger leaf area in treatments with salmon carcasses, but not all species or in all years. Lastly, we also found some positive but varied effects on display size and seed production.

The increase in nitrogen-15 found in all plant species in the two salmon carcass treatments is consistent with other studies examining similar salmon ecosystems [15,21,33]. Studies in Alaska indicate that soil nitrogen-15 may not always reflect available ammonium and nitrate for plant uptake due to confounding soil processes interfering with isotope fractionation [7,22]. This underscores the need to measure variables of biological importance when testing relationships between salmon and riparian or estuarine organisms [14]. Our work provides experimental evidence of an effect of salmon carcass deposition on plant nitrogen-15 under conditions of equal soil chemistry in a single estuarine meadow. However, this difference in ∂N^15^ did not result in a difference in leaf per cent nitrogen among treatments. Terrestrial plants exhibit nutrient ratios and elemental compositions that vary as a function of many factors, such as competition, nutrient limitation, subsidies and species-specific elemental requirements [34]. We might expect to see changes in nutrient ratios across treatments in cases where a nutrient has been provided in great excess, resulting in luxury consumption or excess uptake of that particular nutrient [35,36]. Conversely, plants with naturally high levels of leaf nitrogen tend not to accumulate excess nitrogen in appreciable amounts, and may instead increase their relative growth rate and thus nitrogen levels in their leaves as a whole but not necessarily per unit area [37]. Evidence of luxury consumption is sometimes observed in nitrophilic riparian plants present in salmon ecosystems with high spawning densities [38,39], indicating that high carcass density may result in the short-term resolution of nitrogen limitation in certain cases. In our work, we did not observe variation in %N across treatments, nor evidence of nitrogen luxury consumption despite increases in nitrogen-15. This demonstrates that nitrogen may remain limiting in this particular salt marsh even in the presence of a subsidy that resulted in increases in vegetative and reproductive growth. This is unsurprising given the volume of carcasses and excess nitrogen likely to be needed to achieve luxury consumption in this case. Further work in this system on the availability of specific macro- and micro-nutrients is needed to determine the precise source of the plant trait changes observed.

The addition of a salmon carcass led to larger leaves, especially in yarrow and common red paintbrush. Both plant species are perennial and rhizomatous, though non-flowering vegetative growth is most common in yarrow [40]. As nutrient availability increases, the relative growth rate of a plant's vegetative structures also increases [36]. Additionally, vegetative growth and leaf tissues are more likely to respond to such nutrient pulses than other plant tissues such as roots and stems [41]. No effect was found in silverweed, which produces long stolon runners that root and produce leaf clusters at nodes between the runners [42]. Similarly, the growth habit of Douglas' aster—which also did not exhibit a leaf area response to treatment—is often described as ‘clumping and creeping’ with many inflorescences [40], which may explain the lack of investment in vegetative growth by this species in response to treatment. These results are consistent with another experimental study in the region, which indicated that some plant species benefit from salmon subsidies more than others [21]. In particular, nitrophilic species such as salmonberry (*Rubus spectabilis*) and false lily of the valley (*Maianthemum dilatatum*) dominate metrics of per cent cover in riparian areas near streams with high spawning abundance, a pattern which may be driven by increased vegetative growth in the presence of nitrogen [15]. Forest productivity and tree growth also respond to cyclical variation in salmon abundance and pulses of marine-derived nutrients, a pattern previously observed both experimentally and observationally [23,43,44].

We found varied effects of treatment on floral display, with larger display area in common red paintbrush and Douglas' aster in treatments with salmon across most years. This effect was context-dependent, which reflects prior research on animal subsidies [45], in particular, the quantity, quality, timing and duration of a subsidy, and may interact to yield different effects on recipient ecosystems [45]. Additionally, we found an effect of seaweed deposition on Douglas' aster, which may reflect the addition of common sea wrack micronutrients that are important for plant reproductive structures [24]. Floral display size has important implications for plant fitness, as pollinators may associate display with floral rewards such as pollen or nectar [46]. Paintbrush (*Castilleja)* species are hemiparasites that facultatively parasitize the roots of their hosts [47] and also produce very small flowers amid a large display of petal-like bracts. Thus, the leaf area and floral display area effects in this species are difficult to disentangle. In Douglas' aster, the larger display size is probably an effect of an increase in the number of flowers produced on a single plant, though our analysis calculated total display area as the combination of the size of individual inflorescences and the total number of flowers. Douglas' aster produces compound inflorescences comprising disc and ray flowers, and we find it unlikely that an increase in display size would be simply due to longer ray flower petals alone. Previous research on experimental nutrient additions in alpine and subalpine ecosystems suggests that perennial species' floral traits may take several growing seasons to respond to nitrogen fertilization, and that this effect can vary among years [48–50]. Thus, the year-dependent and varied effects that we observed on floral display are consistent with prior work on plants with similar life histories. Lastly, other unmeasured floral traits such as flowering phenology may respond indirectly to the presence of salmon. Lisi & Schindler [51] found that the timing of salmon spawning indirectly influences the bloom timing of riparian kneeling angelica (*Angelica genuflexa*) via blowfly pollination. If subsidies affect plant phenology, changes in species' competitive interactions and mutualisms with beneficial insects may influence the magnitude of response in plant traits [52]. Thus, we present some of the first evidence that salmon directly influence the floral traits of estuarine plants through fertilization.

The addition of marine-derived nutrients increased seed set in yarrow in the third and final year of the study. This is consistent with prior work on riparian plant species such as salmonberry, which produces a higher abundance of berries per plant on streams with larger returns of chum salmon [19]. Other work on the effect of nitrogen fertilization has shown that seed output may take several seasons of fertilization to respond [50], or may not respond to resource supplementation at all [53]. Burkle & Irwin [48] suggest that perennial plant species may exhibit delayed effects of fertilization, particularly upon seed set. Additionally, our experimental design cannot distinguish between any differences in decomposition rates between salmon carcasses and drift seaweed, which may account for delayed effects of fertilization in some cases. Yarrow is perennial and has high seed output and large, compound inflorescences which produce an order of magnitude more seeds than the inflorescences of the other plant species we studied [40], suggesting that investment in seed set may require more resources than can be integrated in a single season. Despite uptake of salmon-derived nitrogen, silverweed, common red paintbrush and Douglas' aster did not exhibit a seed set response to any treatment in any sampling year. The seed output of hemiparasitic *Castilleja* species can be influenced by their host species via changes in alkaloid uptake and herbivory-pollination feedbacks [47]. The lack of effect in three of the focal species is also consistent with research demonstrating that the presence of salmon-derived nitrogen-15 is often decoupled from meaningful biological consequence for individual organisms [54,55].

In conclusion, we contribute an experimental, causal account of whether and how meta-ecosystem level processes—specifically nutrient transport via aquatic organism migration and mortality—alter the growth and reproduction of multiple species of terrestrial plant. The meta-ecosystem framework suggests that the movement of energy, materials and organisms results in emergent ecosystem properties and functions that are sustained by these movements [56]. These movements can be maintained by environmental and climatic fluctuations [56] or disrupted by human influence, exemplified in fluctuating salmon populations in our study system and their vulnerability to fishing pressure and climate change [57,58]. The ecological threats facing salmon, and more broadly estuarine and stream ecosystems, are not unique [59]. Globally, terrestrial predators are highly reliant on aquatic nutrient subsidies [60], and the nutrient diffusion capacity of large animals in meta-ecosystems to transport nutrients is estimated at less than 10% of historical capacity [4]. Empirically understanding processes that operate in meta-ecosystems allows for a more unified approach to ecosystem-based management amid global change, and a deeper understanding of the consequences of anthropogenic change across scales.

## Data Availability

Data and code may be accessed at https://github.com/adennert/wildflower-traits/. The data are provided in the electronic supplementary material [61].
